# Identification of clinical dermatophyte isolates obtained from Iran by matrix-assisted laser desorption/ionization time-of-flight mass spectrometry

**DOI:** 10.18502/cmm.5.2.1157

**Published:** 2019-06

**Authors:** Mohammad Taghi Hedayati, Saham Ansari, Bahram Ahmadi, Mojtaba Taghizadeh Armaki, Tahereh Shokohi, Mahdi Abastabar, Halil Er, Betil Özhak, Dilara Öğünç, Macit Ilkit, Seyedmojtaba Seyedmousavi

**Affiliations:** 1 Invasive Fungi Research Center, Mazandaran University of Medical Sciences, Sari, Iran; 2 Department of Medical Mycology, School of Medicine, Mazandaran University of Medical Sciences, Sari, Iran; 3 Department of Medical Parasitology and Mycology, School of Medicine, Shahid Beheshti University of Medical Sciences, Tehran, Iran; 4 Department of Medical Laboratory Sciences, Faculty of Paramedical, Bushehr University of Medical Sciences, Bushehr, Iran; 5 Department of Medical Parasitology and Mycology, School of Medicine, Babol University of Medical Sciences, Babol, Iran; 6 Department of Microbiology, Faculty of Medicine, University of Akdeniz, Antalya, Turkey; 7 Department of Microbiology, Division of Mycology, Faculty of Medicine, University of Çukurova, Adana, Turkey; 8 Center of Expertise in Microbiology, Infection Biology and Antimicrobial Pharmacology, Tehran, Iran; 9 Molecular Microbiology Section, Laboratory of Clinical Immunology and Microbiology, National Institute of Allergy and Infectious Diseases, National Institutes of Health, Bethesda, MD, USA

**Keywords:** Dermatophytes, Iran, ITS phylogeny, MALDI-TOF MS

## Abstract

**Background and Purpose:**

Matrix-assisted laser desorption/ionization time-of-flight mass spectrometry (MALDI-TOF MS) is widely used to discriminate among pathogenic microorganisms in clinical laboratories. The aim of this study was to assess the utility of MALDI-TOF MS in the routine identification of clinical dermatophyte isolates obtained from various geographical regions of Iran.

**Materials and Methods:**

A total of 94 isolates, including *Trichophyton* *interdigitale* (n=44), *T. rubrum* (n=40), *T. tonsurans* (n=4), *Microsporum canis* (n=4), and *Epidermophyton floccosum* (*n*=1), were analyzed in this study. The identity of each isolate was determined by polymerase chani reaction amplification and sequencing of the internal transcribed spacer (ITS) region of nuclear-encoded ribosomal DNA and also MALDI-TOF MS. The obtained data by molecular approach were compared with MALDI-TOF MS.

**Results:**

The MALDI-TOF MS led to the identification of 44 (47%) isolates at the species level by generating the spectral score values of ≥ 2.0. However, there was not sufficient agreement between the results of the molecular-based ITS identification methods and MALDI-TOF MS in the species identification of 16 (17%) isolates. The Bruker Daltonics database was also not able to identify protein spectra related to 12 isolates (13%), including *T*.* interdigitale* (n=5), *T*. *rubrum* (n=4), *M*.* canis* (n=2), and *T*.* tonsurans* (n=1).

**Conclusion:**

According to the results, the utility of MALDI-TOF MS as a routine diagnostic tool for the accurate and reliable identification of dermatophytes can be justified whenever the protein spectra of a large set of worldwide clinical isolates are included in the commercial libraries. In addition, MALDI-TOF MS can be alternatively used to construct an in-house reference database.

## Introduction

Dermatophytes are the most successful pathogenic fungi causing superficial mycoses (dermatophytosis, also called ringworm) in both humans and animals worldwide [1]. These fungi are associated with high financial costs for diagnosis, treatment, and prevention [2]. Dermatophytes are categorized into seven genera, including *Trichophyton*, *Microsporum*, *Epidermophyton*, *Lophophyton*, *Paraphyton*, *Nannizzia*, and *Arthroderma* [3].

In the last decades, critical changes have occurred in the epidemiology of dermatophytosis in the different regions of Iran. Nowadays, anthropophilic species, such as *T. mentagrophytes* (*T. interdigitale*), *Epidermophyton floccosum*, and *T. rubrum,* are the main pathogens isolated from patients across the country [4]. It is also estimated that about 5% of the Iranian population carries a dermatophyte infection (e.g., tinea) [5].

The identification of the microbial causative agent at the species level could be important and necessary for the effective treatment of the disease and a way to find the probable source of infection [6]. In the last two decades, the advent of molecular methods has greatly contributed to the rapid diagnosis of infection, coupled with the timely onset of appropriate antifungal therapy [7]. However, there is no consensus on the existing methods for DNA extraction which may result in the achievement of different findings on clinical samples [8]. On the other hand, the use of the phenotypic approach for the identification of the dermatophytes will not usually render consistent results with those of molecular methods. 

A new diagnostic approach using the matrix-assisted laser desorption/ionization-time-of-flight (MALDI-TOF) mass spectrometry (MS)-based strategy has facilitated the effective identification of clinical dermatophyte isolates. Moreover, this approach has led to a drastic reduction in the response times in routine clinical laboratories [9-12]. With this background in mind, the present study was conducted to assess the specificity and practicability of MALDI-TOF MS Biotyper analysis in the identification of clinical dermatophyte isolates obtained from Iran.

## Materials and Methods

***Fungal isolates***

A total of 93 dermatophyte strains were isolated from clinical samples (the skin, nail, and hair). The isolates were cultured on Sabouraud dextrose agar (SDA; BD, Heidelberg, Germany) supplemented with chloramphenicol and cycloheximide. They represented three genera and five species, including *T.* *interdigitale* (n=44), *T*.* rubrum* (n=40), *T*.* tonsurans* (n=4), *Microsporum* *canis* (n=4), and *E. floccosum* (n=1). The dermatophyte isolates were acquired from two cities of Iran, namely Tehran (n=79) and Sari (n=14). The identities of the clinical strains at the species level were confirmed using the PCR-restriction fragment length polymorphism (RFLP) of the ITS region of ribosomal DNA and PCR-sequencing of the calmodulin gene, as described previously [13, 14]. 

***Matrix-assisted laser desorption/ionization time-of-flight mass spectrometry***

The MALDI-TOF MS analysis was conducted at the Department of Microbiology, Medical Faculty of Akdeniz University, Antalya, Turkey.

***Sample preparation***

Formic acid-ethanol protein extraction was performed according to the recommendations of Bruker Daltonics GmbH (Bremen, Germany). Briefly, all isolates were subcultured on SDA and Mycosel agar plates (BD), and then incubated for 48-72 h at 28°C. In the next stage, the physiologically active cells were selected from fresh cultures to be inoculated in 8-ml tubes in a brain-heart infusion medium. The tubes were incubated with shaking at 26°C until sufficient fungal growth was observed. The cultures were then left to stand for approximately 10 min. 

Up to 1.5 ml filamentous fungal sediment was collected from the bottom of the tube and transferred to a 1.5-ml Eppendorf tube. The samples were centrifuged for 2 min at full speed (15,682  *g*). After the removal of the supernatant, 1 ml deionized water was added to the pellet, and the sample was vortex-mixed. The centrifugation was repeated, and then 300 l deionized water was added to the pellet, and the sample was mixed thoroughly. Subsequently, 900 l of 100% ethanol was added to the mixture. 

After the centrifugation of the suspension at 15,682  *g* for 2 min, the supernatant was discarded, and ethanol was completely removed by an additional centrifugation step. In the next stage, the pellet was dried at 26°C, 50 μl of formic acid was added, and the sample was thoroughly vortex-mixed. Next, an equal volume of acetonitrile was added, and the sample was centrifuged again. The supernatant (1 l) was transferred to a MALDI target plate (MSP 96 BC ground steel target; Bruker Daltonics) and allowed to dry at 26°C. Finally, the sample was overlaid with 1 l of saturated  -cyano-4-hydroxycinnamic acid solution (HCCA matrix; Bruker Daltonics). The matrix and sample were allowed to co-crystallize at 26°C.

***Data acquisition***

Analyses were performed using Bruker Microflex instrument, Biotyper software (version 3.0), and database (version 3.1.66; Bruker Daltonics). Microbial identification was accomplished using the linear positive ion mode within a mass range of 2000-20,000 Da. The apparatus was arrayed with a 60-Hz nitrogen laser. For each cumulative spectrum, 240 laser shots were obtained at different positions of a sample spot. 

Bacterial test standard (BTS; Bruker Daltonics) was applied for mass calibration as per manufacturer’s guidelines and showed a typical *Escherichia coli* DH5 alpha peptide and protein profile with additional protein peaks (data not shown). Bruker BTS solution (1 l) was placed on the appropriate spot on the MALDI target plate, overlaid with 1 l of HCCA matrix, and allowed to dry at 26°C. Each analytical phase included a negative extraction control and BTS. 

The species identification-related data generated by MALDI-TOF MS were classified following the manufacturer’s instructions. In this regard, the log-score values of ≥ 2.0, 1.7-1.99, and < 1.7 indicated species identification, represented identification at the genus level, and non-reliable identification (NRI), respectively. 

## Results

***Mass spectrometry***

All 93 clinical dermatophyte isolates were subjected to MALDI-TOF MS analysis for identification at the species level. According to the spectral scores recommended by the manufacturer, 38 (40.9%) and 78 (83.9%) isolates were correctly identified at the species and genus levels, respectively. When lower spectral scores were considered (log-score value of ≥ 1.7 instead of ≥ 2.0), species identification was substantially improved, and 73 (78.5%) clinical dermatophyte isolates were correctly identified. In addition, when no minimum threshold spectral score was set, 87 (93.5%) isolates were correctly identified (Table 1). Overall, 78 (83.9%) strains were identified at the log-score values of 2.0 and 1.7-1.99, while 15 (16.1%) isolates were classed as NRI at the log-score value of < 1.7 using Bruker Daltonics criteria.

**Table:** 

**Species**	**PCR** **(+)**	**MALDI-TOF overview** **(species-level identification)**		**No. isolates identified at the specified Bruker-score values**		
		**Agreement**	**Discordant**	**≥2**	**1.7-1.99**	**<1.7**
*T. interdigitale*	44	40	4 *T. rubrum *(n=3) *T. tonsurans *(n=1)	16^b^	19	6
*T. rubrum*	40	38	2 *T. interdigitale *(n=1) *T. tonsurans *(n=1)	17	19^c^	5^c^
*T. tonsurans*	4	4	-	4^d^	1^e^	1
*M. canis*	4	4	-	1	1	2
*E. floccosum*	1	1	-	0	0	1
Total	93	87	6	38	40	15

Out of the 44 *T. interdigitale* isolates, 15 (34.1%) and 19 (43.2%) species were identified at the log-score values of ≥ 2.0 and 1.7-1.99, respectively. At the same time, *T. interdigitale* isolates were identified as *T. tonsurans* [13 (29.5%) and 15 (34.1%) species at the log-score values of ≥ 2.0 and 1.7-1.99, respectively]*.* On the other hand, *T. tonsurans* isolates were identified as *T. tonsurans *[3 (75%) isolates] or *T. interdigitale* [2 (50%) isolates] at the log-score values of ≥ 2.0. 

Among the representatives of three genera and five species evaluated in the current study, the isolate identification rate was at the highest level for *T. rubrum *isolates (correct identification of 95% of the isolates). *Epidermophyton floccosum* and *M. canis* were identified at the log-score values of < 1.7. Since only a low number of *E. floccosum* and *M. canis* isolates was available, by now, no further conclusion can be made about the utility of MALDI-TOF MS for the identification of two species. 

***Misidentifications and disagreements***

A total of 6 (6.5%) dermatophyte strains (4 *T. interdigitale* strains and 2 *T. rubrum *strains) could not be identified at the species level by using the MALDI-TOF MS Bruker system (Table 1). Most notably, 2 *T. rubrum* strains were misidentified as *T. interdigitale* (n=1) and *T. tonsurans *(n=1) at the log-score values of ≥ 2.0. In addition, 4 *T. interdigitale *strains were misidentified as *T. rubrum* (n=3) and *T. tonsurans *(n=1) at the log-score value of 1.7-1.99, except for one *T. rubrum* strain, the identification of which was made at a log-score value of < 1.7. 

## Discussion

Currently, four different MALDI-TOF MS benchtop platforms are commercialized for the routine identification of fungi in clinical microbiology laboratories. These platforms include the Andromas (Andromas SAS, Paris, France), Axima@Saramis (Shimadzu/AnagnosTec, Duisburg, Germany), Bruker Biotyper (Bruker Daltonics, Bremen, Germany), and Vitek MS (bioMérieux, Marcy l'Etoile, France) systems. However, only the latter two systems have been approved by the US Food and Drug Administration for the identification of bacteria and yeasts [15].

Bruker Daltonics affords the MALDI Biotyper (software and database), and bio-Merieux provides the Vitek MS and SARAMIS (AnagnosTec, Germany) databases referred to as Vitek MS IVD system [16]. Biotyper software generates a log score value range of 0-3 and recommends the scores of 2 and ≥ 1.7 for species-level or genus-level identification, respectively. In addition, a confidence score value of 60% is recommended for species-level identification using the Vitek MS software [15]. 

Our results are in agreement with those of the previous studies showing that the routine diagnosis of dermatophytes at the species level by MALDI-TOF MS is premature at this stage. According to our results, no significant compliance was found between the molecular-based ITS identification method and MALDI-TOF MS for the species identification of 16 (17%) dermatophyte isolates. 

The main concern associated with MALDI-TOF MS-based identification of pathogenic fungi is the similarities of the molecular components, which may cause sister species to be indistinguishable. For each MALDI-TOF MS system, the reference database for the coverage of microbial species is the Achilles' heel of MALDI-TOF MS approach [16]. Regarding this, laboratories are recommended to generate and complement the mass spectra for their locally main species or strains and record them in the reference commercial libraries [17]. 

Until now, only a few studies have addressed the identification of dermatophytes by using MALDI-TOF MS. The present study is the first attempt targetted toward analyzing 93 clinically important dermatophyte isolates obtained from Iran using the MALDI-TOF MS Bruker system (database version 3.1.66). The use of the manufacturer-recommended spectral cutoff scores resulted in relatively poor identification at the genus and species levels. However, the efficiency of species-level identification was substantially improved when the log score threshold was reduced. These observations are in agreement with the findings of Karabicak et al. [18] and Theel et al. [19] who showed that lowering spectral scores increases the precision of identification. 

The reported dermatophyte identification rates by MALDI-TOF MS varied from 13.5% to 100% for dermatophytes [20]. However, the efficacy of this technique mainly relies on the standard manufacturer-supplied library or a supplemented manufacturer library [18-20]. The use of these standard libraries underpins the reported variable findings as library expansion improves accurate dermatophyte identification by MALDI-TOF MS [18-20]. Effective identification of dermatophyte species relies on the number and variety of isolates available in the reference spectral library [18-22]. 

Because of the major limitations of the commercial reference spectral libraries, in-house reference libraries have been introduced in most previous studies [18-25]. Therefore, a commercial reference spectral library should be developed to encompass a wide range of different strains. However, in the present study, we only used a commercial reference spectral library. It is likely that an in-house library would allow an improved correct identification of the Iranian dermatophyte isolates evaluated.

According to the molecular epidemiological studies, *T. interdigitale *and* T. rubrum* are the most common dermatophytes isolated in Iran [4, 13, 26, 27]. In the present study, 38 (95%) and 40 (90.9%) *T. rubrum* and *T. interdigitale *isolates, respectively, were correctly identified by MALDI-TOF MS. In medical mycology laboratories, the differentiation between *T. rubrum *and *T. interdigitale* based on morphological characteristics is challenging. For example, in a study performed by Ahmadi et al. [28], out of the 94 molecularly identified isolates of dermatophytes, 80.8% of *T. rubrum *were recognized as *T. interdigitale* (75.5%), *E. floccosum* (2.1%), *M. canis* and *T. verrucosum *(1.06%), and *T. tonsurans* (1.06%) by morphological testing. 

The misidentification might stem from a number of issues, including morphological resemblance of the two species on SDA; similar colony texture; scattered micro-conidia; and orange-yellow or melanoid pigment production by some isolates [28]. Since *T. rubrum *and *T. interdigitale *are the most common clinical dermatophyte strains isolated [29], MALDI-TOF MS might aid their accurate identification. 

## Conclusion

In conclusion, our study demonstrated that MALDI-TOF MS is a rapid, inexpensive, reliable, and advanced method. This might comprise a good alternative for the laboratory identification of dermatophytes. Given that currently available commercial spectral libraries are not ideal for routine dermatophyte identification, the development of an in-house reference library seems necessary to increase the credibility of this technique, which warrants further investigation. 
